# Exercise Training Preserves Myocardial Strain and Improves Exercise Tolerance in Doxorubicin-Induced Cardiotoxicity

**DOI:** 10.3389/fcvm.2021.605993

**Published:** 2021-04-01

**Authors:** Igor L. Gomes-Santos, Camila P. Jordão, Clevia S. Passos, Patricia C. Brum, Edilamar M. Oliveira, Roger Chammas, Anamaria A. Camargo, Carlos E. Negrão

**Affiliations:** ^1^Faculdade de Medicina, Heart Institute (InCor), Hospital das Clínicas, Universidade de São Paulo, São Paulo, Brazil; ^2^School of Physical Education and Sport, Universidade de São Paulo, São Paulo, Brazil; ^3^Faculdade de Medicina, Cancer Institute of the State of São Paulo (ICESP), Hospital das Clínicas, Universidade de São Paulo, São Paulo, Brazil; ^4^Centro de Oncologia Molecular, Hospital Sírio-Libanês, São Paulo, Brazil

**Keywords:** exercise, cardiac function, speckle tracking, doxorubicin, cardiotoxicity, fatigue

## Abstract

Doxorubicin causes cardiotoxicity and exercise intolerance. Pre-conditioning exercise training seems to prevent doxorubicin-induced cardiac damage. However, the effectiveness of the cardioprotective effects of exercise training concomitantly with doxorubicin treatment remains largely unknown. To determine whether low-to-moderate intensity aerobic exercise training during doxorubicin treatment would prevent cardiotoxicity and exercise intolerance, we performed exercise training concomitantly with chronic doxorubicin treatment in mice. Ventricular structure and function were accessed by echocardiography, exercise tolerance by maximal exercise test, and cardiac biology by histological and molecular techniques. Doxorubicin-induced cardiotoxicity, evidenced by impaired ventricular function, cardiac atrophy, and fibrosis. Exercise training did not preserve left ventricular ejection fraction or reduced fibrosis. However, exercise training preserved myocardial circumferential strain alleviated cardiac atrophy and restored cardiomyocyte cross-sectional area. On the other hand, exercise training exacerbated doxorubicin-induced body wasting without affecting survival. Finally, exercise training blunted doxorubicin-induced exercise intolerance. Exercise training performed during doxorubicin-based chemotherapy can be a valuable approach to attenuate cardiotoxicity.

## Introduction

Doxorubicin (Doxo) is an antineoplastic agent widely used to treat various cancer types over the last decades. Its clinical applications, however, are hampered by several adverse side effects. It affects healthy organs and systems as well (1), by inducing cardiotoxicity (2) and contributing to tiredness and exercise intolerance, also reported as cancer-related fatigue (3, 4).

Previous studies demonstrated that Doxo-induced cardiotoxicity is a dose-dependent phenomenon, defining safety ranges for Doxo in preventing heart failure (HF) (5–7). However, Doxo treatment remains prevalently associated with ventricular dysfunction, characterized by a reduction of left ventricular (LV) ejection fraction (EF) > 10% from baseline or displaying LVEF < 50% (5, 7). Although clinically overt cardiotoxicity occurs in ~6% of cancer patients, at least one in seven patients will present subclinical cardiotoxicity, and 1 out of 10 will experience an adverse cardiovascular event (8). This borderline impairment of ventricular function may not reach the minimum guideline-levels for starting a pharmacological intervention during cancer treatment, but it might account for the high incidence of cardiovascular morbidity and mortality observed in previously treated patients, even several years after defeating cancer (9, 10). Hence, finding strategies to prevent Doxo-related adverse effects are highly desirable.

Exercise training (ExTr) has been suggested as a non-pharmacological approach against Doxo-induced cancer-related fatigue and cardiotoxicity (1, 11–15). Several studies have consistently demonstrated a protective role of ExTr when performed before chemotherapy in preclinical models. However, it remains unknown whether ExTr performed *concomitantly* with Doxo-based treatment prevents cardiotoxicity (15). Considering the adherence to ExTr programs in cancer patients declines as the dose of ExTr intensity increases (16), we tested the hypothesis that a low-to-moderate intensity aerobic ExTr program during Doxo treatment would prevent cardiotoxicity and exercise intolerance.

## Methods

### Animal Care and Experimental Protocol

All animals care and procedures were conducted following the National Council for the Control of Animal Experimentation (CONCEA) and approved by the Institutional Scientific Committee of the Heart Institute (InCor-HCFMUSP) and the Ethics Committee on Animal Use (CEUA) of the University of São Paulo Medical School (FMUSP). Eight-week-old male C57BL/6J mice were enrolled in the study. Mice were evaluated for exercise capacity and LV structure and function and then assigned into three groups: Control (no exercise, saline-treated, *n* = 12), Doxo (no exercise, treated with doxorubicin, *n* = 20) and Doxo + ExTr (exercise-trained, treated with doxorubicin, *n* = 20). They were kept in a temperature-controlled room with a 12:12-h light-dark cycle and free access to a standard diet and water. The animals were euthanized by cervical dislocation under deep anesthesia. The heart was dissected to separate right ventricle (RV), left ventricle (LF), and septum, allowing the assessment of RV hypertrophy using Fulton's index (RV/LV + S). Heart and Lungs were weighted for determination of absolute (wet tissue, in mg) or relative (normalized by tibia length, mm) mass. Heart samples (LV) were then snap-frozen in liquid nitrogen and stored at −80°C for biochemical and histological analysis.

### Echocardiography

The LV evaluations were performed using a preclinical ultrasound system (Vevo 2100 Imaging System, Visual Sonics, Canada), and mice were sedated with 5% isoflurane and kept under anesthesia by continuous administration of 1–1.5% of isoflurane with 1 L/min 100% O_2_ to maintain light sedation throughout the procedure. Transthoracic echocardiography was performed using a 40-MHz transducer positioned on the mice shaved chest with contact gel, and images were stored on cine loops at the time of the study using a digital Two-dimensional B mode with short-axis views at the level of the papillary muscle. LV dimensions were measured in the B-mode, in the 2-dimensional parasternal long-axis view. All the analyses were conducted according to the American Society of Echocardiography guidelines (17). Strain analysis was based on speckle tracking technique (18), which uses acoustic backscatter on 2D grayscale ultrasound images (two-dimensional cinematic images) as tissue marker, allowing the quantification of myocardial deformation along the longitudinal, radial, and circumferential axes, according to myocardial fiber orientation. The parasternal long-axis view provided a longitudinal and radial strain and strain rate. Circumferential and radial strain and strain rates were obtained from the parasternal short-axis view. Both long- and short-axis views are divided automatically into six segments for speckle tracking throughout the cardiac cycle and their average provided global (longitudinal, circumferential, and radial) parameters.

### Determination of Exercise Capacity and Prescription of Exercise Training

Maximal running capacity tests were performed at baseline and at the end of the experimental protocol on a mouse running treadmill, providing parameters of exercise tolerance (running time, in s and running performance, in J) and proper exercise prescription, as described previously (19). The ExTr program consisted of a continuous, low-to-moderate aerobic ExTr (~40–50% of maximal exercise capacity at 0% grade, 40 min per session, 4 days per week for 5 weeks).

### Doxorubicin Treatment

Doxorubicin was obtained from the Cancer Institute, University of São Paulo Medical School (ICESP-HCFMUSP) pharmacy. Doxo was administered once a week via intraperitoneal injections of 5 mg/kg for 5 weeks (20). Saline groups received 0.9% saline injection on the same volume of Doxo-treated groups (0.1 mL). Mice were not subjected to ExTr on the day of Doxo or saline injections, neither for the following 2 days.

### Western Blot

Protein expression levels were analyzed by immunoblotting. Frozen samples from LV were homogenized in Tris-HCl buffer (100 mM Tris-HCl, 50 nM NaCl, 1% Triton, pH 7.4) and protease/phosphatase inhibition cocktail (1:100, Sigma-Aldrich, USA). After centrifugation (10,000 × g, 4°C, 10 min), we recovered the supernatant and added a loading buffer (Laemmli 1:1, Sigma-Aldrich, USA). Samples (30 μg) were then transferred to SDS-PAGE in 10% acrylamide gels and submitted electrophoresis and then electrically transferred to a nitrocellulose membrane (BioRad Biosciences, USA). The membranes were incubated in a blocking solution (5% BSA, 10 mM Tris-HCl, pH 7.6, 150 mM NaCl, e 0.1% Tween 20) for 3 h in room temperature, then overnight in 4°C with primary antibodies. The primary antibodies binding was detected using secondary antibodies with peroxidase activity, reaction detected by chemiluminescence (Amersham Biosciences, USA), and visualized by autoradiography. Mouse monoclonal anti-GATA4 (Santa Cruz sc-25310, 1:500), rabbit polyclonal anti-SOD1[Cu-Zn] (Abcam ab16831, 1:2,000), rabbit polyclonal anti-SOD2[Mn] (Abcam ab13533, 1:5,000) and mouse monoclonal anti-SOD3[EC] (Abcam ab80946, 1:1,000) total expression levels were normalized by a housekeep gene (mouse monoclonal anti-ß-actin, Santa Cruz sc-47778), and rabbit monoclonal anti-phospho-ERK1/2 [Thr202/Tyr 204] (Cell Signaling #4377, 1:1,000) as ratio to total rabbit monoclonal anti-ERK1/2 (Cell Signaling #4695, 1:1,000). We also analyzed GATA4 in the cytosolic and nuclear fractions. The cytosolic and nuclear fractions were obtained by an extraction kit (NER-PER, ThermoFisher Scientific, EUA). We used Image-J (National Institute of Health, USA) software to quantify immunoblots density.

### Histological Analysis

Cross-sectional rings of the mid-left ventricles were frozen in nitrogen-cooled isopentane. Then, samples were serially sliced into 10-μm cross-sections from the proximal to the distal region using a cryostat (Micron HM505E, Zeiss, Germany). The fiber cross-sectional area (CSA) was evaluated in transverse cardiomyocytes, stained with Griffonia simplicifolia lectin (1 h at room temperature), and imaged at 40x magnification. CSA of adjacent cardiomyocytes from at least three slides per mice, *n* = 2 mice per group were pooled, averaging ~300 cardiomyocytes per group. Collagen deposition was quantified in cardiac sections stained with Picrossirius Red, and imaged under 40x objective. The images were thresholded, and the interstitial collagen fractional area was averaged from at least five fields per slide, *n* = 5 mice per group, and expressed as a percentage. Images were acquired using a Leica Quantimet 520 (Cambridge Instruments, UK), and analyzed with Image J (National Institute of Health, USA).

### Statistical Analysis

Results are expressed as Mean ± SEM. Statistical analyses were performed using GraphPad Prism software. Kaplan-Meier plots were generated and a log-rank test performed to compare exercise time tolerance curves. Endpoints were compared by one-way ANOVA with Tuckey's multiple comparison tests or Student *t*-test to detect group differences. We used linear regression for correlative analysis. In all cases, we considered as significant a *P* ≤ 0.05.

## Results

### Doxo-Induced LVEF Reduction Is Not Prevented by ExTr

**Figure 1:**
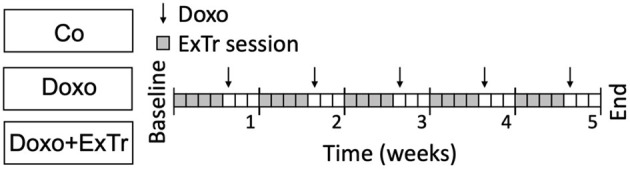
Experimental design. Doxorubicin was administered weekly (5 mg/kg i.p. for 5-wk), concomitantly with low-to-moderate intensity aerobic exercise training (40–50% maximal exercise capacity for 40 min, 4 days per week), or follow-up. Co, control (*n* = 12); Doxo, doxorubicin (*n* = 20); Doxo + ExTr, doxorubicin + exercise training (*n* = 20); ExTr, exercise training.

**Figure 2:**
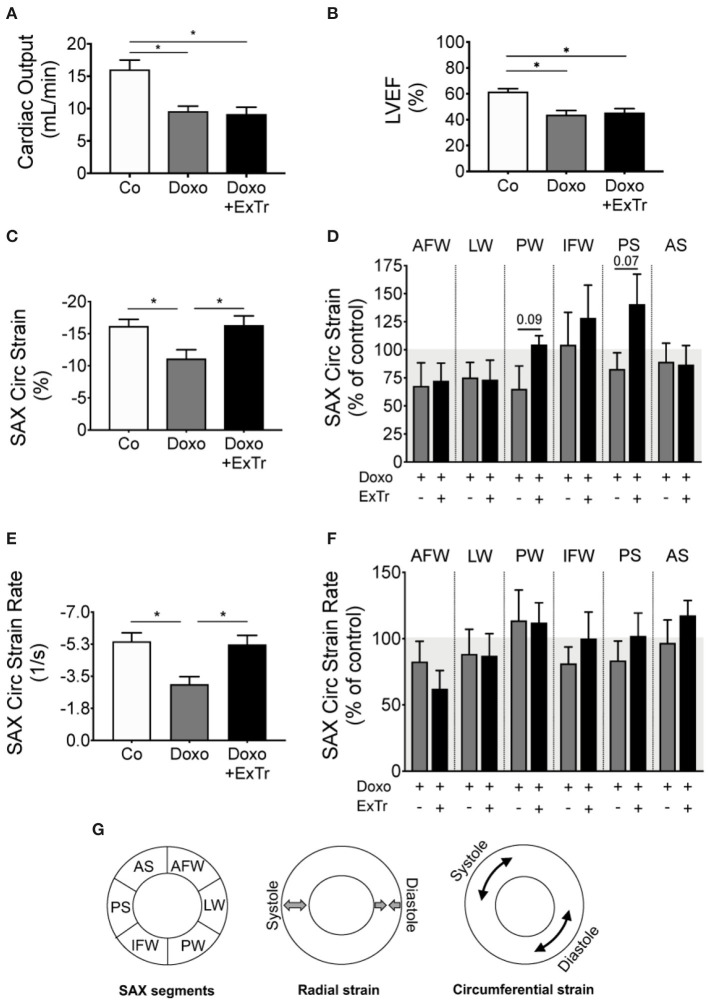
Exercise training alleviates Doxo-induced cardiac dysfunction. Doxo impairs CO **(A)** and LVEF **(B)**. However, speckle tracking showed ExTr preserved SAX cardiac contractility, both in the Circ strain **(C,D)** and strain rate **(E,F)**, expressed as a final vector from strain **(B)** and strain rate **(D)** of different cardiac segments **(D,F)**. AFW, anterior free wall; AS, anterior septum; CO, cardiac output; Circ, circumferential; IFW, inferior free wall; LW, lateral wall; LVEF, left ventricular ejection fraction; PS, posterior septum; PW, posterior wall; SAX, short axis). Conceptual analysis of speckle tracking on **(G)**. *n* = 6–11 per group. Group abbreviations as in Figure 1. ^*^P ≤ 0.05.

We first conducted a maximal exercise capacity test to properly prescribe ExTr, as well as echocardiography to set baseline cardiac parameters (displayed in Table 1). We then applied a Doxo treatment previously established (Figure 1) (20), exposing mice to an accumulated dose of 25 mg/kg. Echocardiography confirmed Doxo-induced cardiotoxicity, characterized by reduced cardiac output (CO) and LVEF (Table 2 and Figures 2A,B). LVEF reduced 26% with Doxo alone, and 22% under Doxo + ExTr in comparison to Control (Figure 2B). Under conventional echocardiography, ExTr was not able to prevent Doxo-induced LV dysfunction expressed by CO and LVEF (Figures 2A,B), LV fractional shortening (FS), and stroke volume (SV) (Table 2).

**Table 1:** Physical characteristics, ventricular morphology and function, and effort tolerance at baseline.

	**Co**	**Doxo**	**Doxo + ExTr**	***P*-value**
Bodyweight, g	23.6 ± 0.43	23.3 ± 0.46	23.1 ± 0.2	0.66
HR, bpm	437 ± 24	387 ± 26	365 ± 31	0.93
**Left ventricular morphology**				
LVEDD, mm	3.61 ± 0.09	3.60 ± 0.08	3.58 ± 0.11	0.49
LVESD, mm	2.44 ± 0.14	2.54 ± 0.15	2.43 ± 0.22	0.89
IVS, mm	0.77 ± 0.01	0.76 ± 0.03	0.79 ± 0.02	0.63
LVPW, mm	0.69 ± 0.03	0.64 ± 0.02	0.69 ± 0.02	0.32
**Systolic function**				
LVEF, %	60.1 ± 4.92	56.0 ± 5.29	59.6 ± 7.05	0.61
LVFS, %	32.4 ± 3.29	29.5 ± 3.55	32.8 ± 4.82	0.49
LVEDV, μL	55.3 ± 3.61	54.9 ± 3.03	54.4 ± 3.63	0.81
LVESV, μL	22.3 ± 3.27	24.4 ± 3.38	23.3 ± 4.83	0.58
CO, mL/min	14.6 ± 1.80	11.4 ± 1.05	11.4 ± 1.72	0.17
SV, μL	33.0 ± 3.25	30.4 ± 2.96	31.1 ± 3.26	0.85
**Diastolic function**				
Mitral E velocity, mm/s	478 ± 57.9	574 ± 46.5	481 ± 59.9	0.99
Mitral A velocity, mm/s	200 ± 36.8	269 ± 39.1	201 ± 20.4	0.49
Mitral E/A ratio	2.78 ± 0.44	2.31 ± 0.32	2.20 ± 0.20	0.47
**Exercise tolerance**				
Running distance, m	665 ± 41.3	603 ± 52.3	621 ± 27.5	0.66
Running performance, J	154 ± 11.6	140 ± 11.8	143 ± 6.14	0.98

*n = 8–11 Control, n = 8–9 Doxo, n = 7–9 Doxo + ExTr. One-Way ANOVA*.

*CO, cardiac output; HR, heart rate; IVS, interventricular septal wall thickness; LVEDD, left ventricular end-diastolic diameter; LVEDV, left ventricular end-diastolic volume; LVESV, left ventricular end-systolic volume; LVEF, left ventricular ejection fraction; LVESD, left ventricular end-systolic diameter; LVPW, left ventricular posterior wall thickness; LVSF, left ventricular stroke fraction; SV, stroke volume*.

**Table 2:** Physical characteristics, ventricular morphology, and function after 5 weeks of saline or doxorubicin treatment.

	**Co**	**Doxo**	**Doxo + ExTr**	***P*-value**
Bodyweight, g	25.3 ± 0.71	21.3 ± 0.61^*^	19.2 ± 0.43^*^	< 0.001
HR, bpm	414 ± 13	416 ± 30	389 ± 26	0.65
Wet cardiac mass, mg	102.1 ± 2.63	75.2 ± 2.48^*^	81.1 ± 4.75^*^	< 0.001
Wet LV, mg	81.7 ± 2.19	62.0 ± 2.31^*^	65.9 ± 3.64^*^	< 0.001
Wet lung, mg	209.1 ± 9.17	157.3 ± 12.6^*^	170.8 ± 12.4^*^	0.007
**Left ventricular morphology**				
LVEDD, mm	3.81 ± 0.16	3.59 ± 0.13	3.51 ± 0.12	0.33
LVESD, mm	2.57 ± 0.14	2.83 ± 0.14	2.74 ± 0.13	0.44
IVS, mm	0.82 ± 0.04	0.71 ± 0.03	0.73 ± 0.03	0.16
LVPW, mm	0.71 ± 0.03	0.67 ± 0.03	0.67 ± 0.02	0.59
**Systolic function**				
LVFS, %	32.8 ± 1.66	21.3 ± 1.93^*^	22.3 ± 1.72^*^	< 0.001
LVEDV, μL	63.9 ± 6.00	54.7 ± 4.64	52.2 ± 4.32	0.25
LVESV, μL	25.2 ± 3.35	31.2 ± 3.60	29.0 ± 3.56	0.48
SV, μL	38.7 ± 3.22	23.5 ± 2.05^*^	23.2 ± 1.52^*^	< 0.001
**Diastolic function**				
Mitral E velocity, mm/s	431 ± 32.5	433 ± 41.1	390 ± 38.6	0.66
Mitral A velocity, mm/s	227 ± 23.6	201 ± 65.9	208 ± 31.5	0.89
Mitral E/A ratio	2.01 ± 1.85	3.35 ± 1.12	2.16 ± 0.39	0.27
**Speckle-tracking**				
LAX Longitudinal strain, %	−11.8 ± 1.57	−17.9 ± 2.50	−15.5 ± 2.13	0.75
LAX Longitudinal SR, 1/s	−5.1 ± 1.04	−6.67 ± 1.14	−7.46 ± 0.89	0.18
LAX Radial strain, %	19.4 ± 3.13	30.8 ± 4.83^(0.09)^	30.2 ± 2.64^(0.09)^	0.04^*^
LAX Radial SR, 1/s	6.57 ± 1.45	8.87 ± 1.25	9.01 ± 0.74	0.28
SAX Radial strain, %	32.6 ± 2.96	25.9 ± 3.52	26.3 ± 2.97	0.23
SAX Radial SR, 1/s	8.58 ± 0.97	7.82 ± 0.50	8.36 ± 0.72	0.84

*n = 7–12 Control, n = 6–9 Doxo, n = 6–11 Doxo+ExTr*.

* *P ≤ 0.05 vs. Control by One-Way ANOVA and Tukey's multiple comparisons test*.

*CO, cardiac output; HR, heart rate; IVS, interventricular septal wall thickness; LAX, long axis view; LVEDD, left ventricular end-diastolic diameter; LVEDV, left ventricular end-diastolic volume; LVESV, left ventricular end-systolic volume; LVEF, left ventricular ejection fraction; LVESD, left ventricular end-systolic diameter; LVPW, left ventricular posterior wall thickness; LVSF, left ventricular stroke fraction; SAX, short axis view; SR, strain rate; SV, stroke volume; TL, tibia length*.

### The Myocardial Circumferential Strain Is Preserved With ExTr

Speckle tracking analysis indicated that Doxo significantly affected the circumferential SAX strain (Figure 2C) and strain rate (SR) (Figure 2E). Those are quantifications of how much the myocardium has deformed in every cycle and at what velocity, respectively. They represent the vector of strain and SR of six regional segments (Figures 2D,F, schematic localization of each segment in Figure 2G). Cardiac strain analysis showed that ExTr prevented the deleterious effects of Doxo in the SAX circumferential strain and SR (Figure 2). ExTr brought the posterior wall (PW), inferior free wall (IFW), and posterior septum (PS) toward normal ranges, therefore normalizing global parameters of circumferential strain and SR in SAX (Figures 2C–G). This suggests a benefic effect of ExTr on regional myocardial contractility.

### ExTr Prevents Doxo-Induced Cardiopulmonary Atrophy

**Figure 3:**
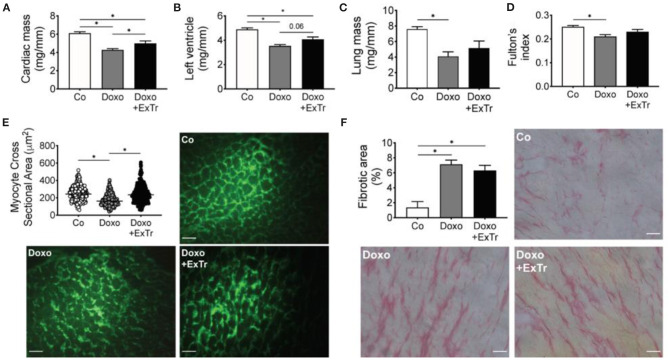
ExTr alleviates Doxo-induced cardiopulmonary atrophy. Doxo induces cardiac **(A)**, left ventricle **(B)**, and lung **(C)** mass atrophy (normalized by tibia length and expressed in mg/mm), which is alleviated by ExTr. Fulton's index (RV/LV + S) reduces with Doxo and is partially preserved by ExTr **(D)**. ExTr prevents cardiomyocyte atrophy induced by Doxo **(E)**. Doxo increases cardiac collagen deposition that is not prevented by ExTr **(F)**. Group abbreviations as in Figure 1. *N* = 8–11 **(A–D)**, >500 cardiomyocytes pooled from *n* = 2–3 **(E)** and *n* = 5 **(F)** per group. ^*^P ≤ 0.05.

**Figure 4:**
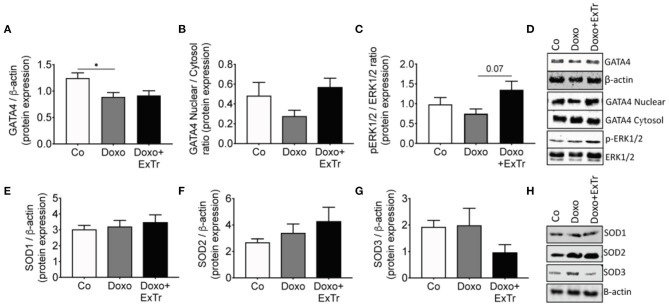
Molecular markers of Doxo-induced cardiotoxicity. Cardiac protein expression of GATA4 analyzed by densitometry and normalized against β-actin **(A)** and its nuclear/cytosol ratio **(B)**; the ratio of pERK-to-total-ERK **(C)** and representative blots **(D)**; protein levels of the antioxidant enzyme superoxide dismutase isoforms 1 **(E)**, 2 **(F)**, or 3 **(G)** and its representative blots **(H)**. GATA4, GATA binding protein 4; ERK, extracellular signal-regulated kinases; pERK, phosphorylated ERK; SOD, superoxide dismutase. Group abbreviations as in Figure 1. *n* = 5–8 per group. ^*^P ≤ 0.05.

Pathological analysis indicated Doxo induced atrophy of cardiac and pulmonary tissue (Table 2 and Figure 3). Cardiac mass (Figure 3A), left ventricle (Figure 3B), and lung (Figure 3C) relative mass were reduced with Doxo, as well as Fulton's index (Figure 3D). ExTr partially alleviated Doxo effects on these parameters. Histological analysis showed Doxo reduces the cross-sectional area of cardiomyocytes (Figure 3E), further confirming cardiac atrophy. Importantly, ExTr sustained a normal cardiomyocyte area. Doxo also induced a ~3-fold increase in myocardial fibrosis that was not prevented by ExTr (Figure 3F). Cardiac levels of the GATA4, a transcription factor that is associated with compensatory response to cardiac injury in the adult heart (21), was significantly reduced under Doxo treatment. ExTr did not restore GATA4 expression, but it attenuated Doxo effects (Figure 4A). As the activity of GATA4 is mainly coordinated by its translocation and nuclear activation, we determined the protein expression levels specifically in the nucleus and in the cytosol. Despite a visual trend toward the reduction of nuclear translocation by Doxo and restoration by ExTr, no statistical significance was observed in the differential expression of GATA4 (Figure 4B). ExTr trend to increase phosphorylated-to-total ERK ratio to Doxo group (Figure 4C). No differences were found in the protein expression levels of the different isoforms of superoxide dismutase (SOD) enzymes (Figures 4E–H).

### ExTr Exacerbates Doxo-Induced Body Wasting

**Figure 5:**
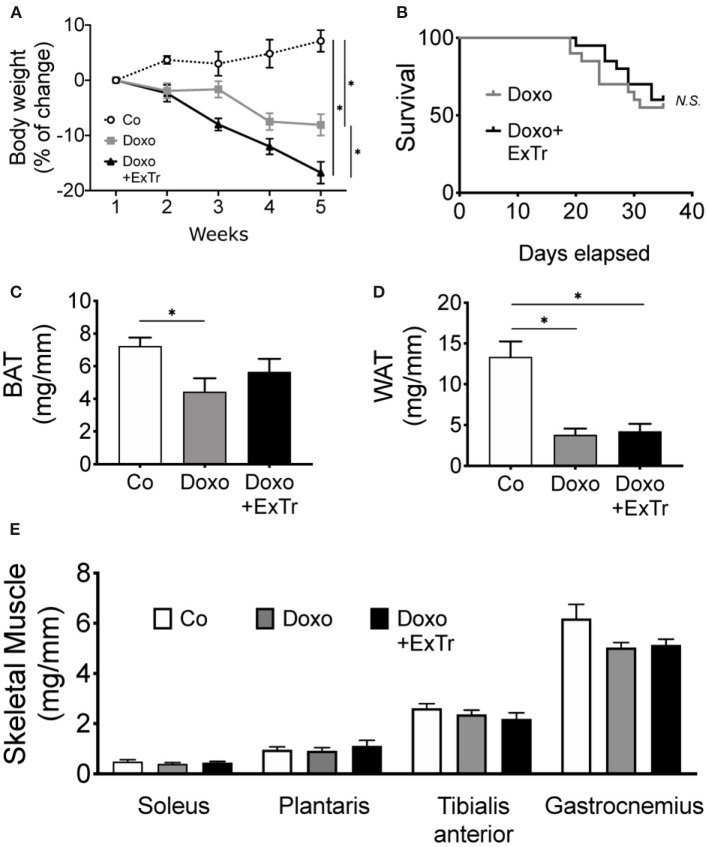
ExTr exacerbates Doxo-induced body wasting. Doxo induces body wasting, which is exacerbated by ExTr **(A)**, although without negatively affecting survival **(B)**. Doxo affects fat depots mass, reducing both BAT **(C)** and WAT **(D)** adipose tissue. ExTr attenuates BAT mass wasting, but not WAT. No changes were found in skeletal muscle mass **(E)**. BAT, brown adipose tissue; WAT, white adipose tissue. Group abbreviations as in Figure 1. *n* = 12–20 per group **(A,B)** or *n* = 8–10 **(C–E)**. ^*^P ≤ 0.05.

Doxo induced body wasting, and ExTr exacerbated it (Figure 5A). However, ExTr did not affect survival under doxo treatment (Figure 5B). One explanation is ExTr might have increased caloric expenditure, as we did not observe changes in food intake among groups (data not shown). This is also supported by the fact that ExTr partially preserved brown adipose tissue (BAT) (Figure 5C), but not white (WAT) fat depots mass (Figure 5D), reduced under Doxo treatment. No changes were observed in skeletal muscle mass (Figure 5E).

### ExTr Blunts Effort Intolerance Induced by Doxo Treatment

**Figure 6:**
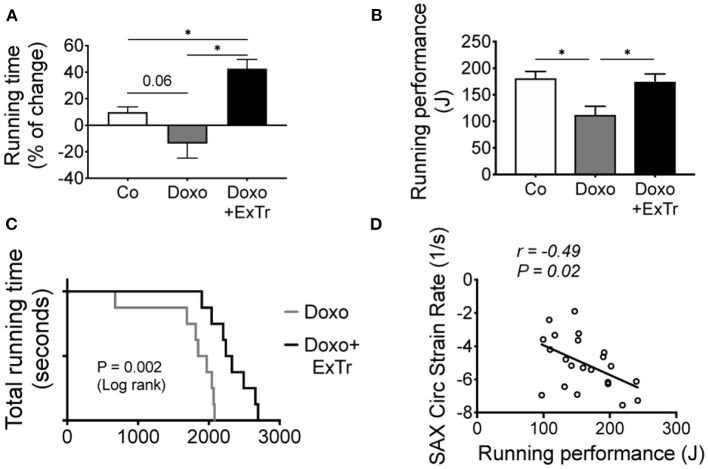
ExTr blunts Doxo-induced effort intolerance. Effort tolerance, determined maximal running capacity, shows Doxo total running time from Baseline trend to impair **(A)**, and running performance **(B)** and total running time **(C)** were reduced. Interestingly, the better the cardiac contractility (strain rate), the higher is exercise capacity **(D)** among Doxo-treated groups. J, joule; SAX, short axis. Group abbreviations as in Figure 1. *n* = 10–12 per group. ^*^P ≤ 0.05.

We quantified running capacity (Figure 6), as it predicts maximal oxygen uptake and cardiopulmonary performance (22). We observed a strong trend toward the reduction of running distance under Doxo treatment, in comparison to baseline levels (Figure 6A). Running performance (a more sensitive method that takes bodyweight in consideration) showed an almost 40% decline induced by Doxo in comparison to the Co group (Figure 6B). ExTr was able to increase the distance covered from baseline in 37%, completely preventing Doxo-induced impaired running performance (Figures 6A,B, respectively). Comparing Doxo-treated groups alone, all mice subjected to ExTr sustained exercise for significantly longer periods than Doxo-treated only (Figure 6C). Interestingly, we found a significant negative correlation between running performance and SAX circumferential strain rate (Figure 6D).

## Discussion

Anthracycline-based chemotherapies are widely used to treat oncologic patients, but it is associated with strong adverse effects, as cardiotoxicity and cancer-related fatigue. As standard care improved, patients are now more likely to survive cancer (23). Hence, strategies to alleviate treatment side effects, prevent the development of co-morbidities, and enhance the quality of life are needed.

Although the role of ExTr to prevent Doxo-induced cardiac dysfunction has been consistently demonstrated in preclinical models, the vast majority of these studies started weeks or even months before either an acute (bolus administration of an accumulated dose of Doxo) or chronic treatment (fractioned dose along time) [find an overview in (24)]. This approach may be relevant to investigate potential mechanisms, but it might not reflect the pathophysiological events observed in the clinical practice. That because a cytotoxic chemotherapy treatment usually starts as soon as possible after diagnosis, limiting the available window for ExTr pre-conditioning.

Some authors analyzed ExTr concomitantly with Doxo, by applying short-term protocols. However, a short treatment period does not allow enough time to the deterioration of systolic function, as expected with chronic Doxo administration (25). Other accessed ventricular function after a wash-out period (12, 20). However, this approach is more likely to favor recovery (26) and, hence, does not necessarily reflect Doxo-induced cardiotoxicity during cancer treatment. The same problem occurs to studies modeling rehabilitation, with ExTr beginning once the cardiomyopathy is established (11), rather than recapitulating a cancer survivorship context. We designed this study to model an ExTr program that started *concomitantly* with chronic cytotoxic treatment with Doxo-based chemotherapy. The synergistic effect of ExTr on the anti-tumoral activity of Doxo has been previously demonstrated (25, 27). Moreover, tumors *per se* can induce ventricular dysfunction (28–30). Therefore, we decided to study non-tumor bearing mice.

The pathophysiology of Doxo-induced cardiotoxicity is complex, and several distinct mechanisms have been established [see (5) for concise review]. On the other hand, chronic and late effects are seemingly related to transcriptional activity abnormalities (20), and the transcription factor GATA4 has been mechanistically implicated (2, 31). In postnatal hearts, GATA4 is essential for cardiomyocyte survival after injury or stress (21). Hence, by inhibiting GATA4, Doxo blunts the cardiac capacity of responding to injury, triggering apoptosis, and autophagy, therefore leading to cardiac atrophy. So far, there is no commercially available pharmacological treatment targeting GATA4. However, studies from our group and others (32, 33) demonstrated that ExTr modulates GATA4, potentially indicating a mechanism for ExTr-induced cardioprotection to Doxo.

ExTr imposes a higher cardiac workload, inducing angiogenesis and hypertrophy of cardiomyocytes, and GATA4 triggers this physiological process (21, 32, 33). However, overexpression of GATA4 is also associated with decompensated cardiac hypertrophy, as it occurs in neuroendocrine overactivation-induced HF (33). On the other hand, in diabetes-induced cardiac atrophy, a similar phenotype observed in response to Doxo, GATA4 activity is reduced (32). Importantly, ExTr brings the activity of GATA4 back to normal physiological ranges in both situations. Hence, one would expect that, if ExTr prevents Doxo-induced cardiotoxicity, it would be by sustaining the transcriptional activity of GATA4 (24). In our study, ExTr alleviated but was not able to restore the expression levels of GATA4 (Figures 4A,B,D). This might explain why ExTr did not prevent Doxo-induced reduction of LVEF (Figure 2B). We cannot exclude the possibility that ExTr favors the regain of ventricular function over time, perhaps even rescuing GATA4 activity. The main explanation for the attenuated GATA4 response to ExTr relies on DNA machinery to function. Doxo is a non-selective inhibitor of topoisomerase (20, 34), an essential enzyme for cell replication and DNA promoting activity. Once topoisomerase is inhibited, it impairs the binding of a transcription factor to the promoter region (34), leading to cardiotoxicity (20). It is likely that, in an “acute” phase [i.e., right after the treatment termination (35)], Doxo still inhibits GATA4 transcriptional activity, preventing ExTr to exert a major early-phase protective effect.

A great deal of studies implicated the increased oxidative stress (ROS) in Doxo-induced cardiotoxicity (36). ExTr is known to reduce cardiac ROS in cardiovascular diseases (13). Here, we assessed the protein levels of SOD as surrogate markers of anti-oxidative capacity, as these are the main enzymes to convert superoxide radicals into hydrogen peroxide and oxygen. As in previous reports, Doxo did not change the expression of SOD in the heart (12). However, in our study, ExTr alone was not able to enhance SOD activity as well. We did not directly measure cardiac ROS generation. Hence we cannot rule out the possibility that ExTr alleviates ROS, which could also explain the modest ExTr effect on myocardial contractility.

Doxo provoked myocardial strain and strain rate abnormalities, analyzed by the speckle tracking technique (37–40). This technique has demonstrated superiority to detect cardiac abnormalities—especially subclinical—over the conventional echocardiography, as myocardial strain reduction is frequent in cancer patients and precedes LVEF reduction that cannot be anticipated by other parameters (37). In our study, we observed a significant impairment of circumferential strain and strain rate in the SAX, which was abrogated by ExTr. We further analyzed each of the six segments that compose the final strain vector (Figures 2C–F). As can be observed, ExTr trends toward bringing reduced myocardial strain under Doxo back to normal ranges. Surprisingly, Doxo did not change LV longitudinal strain or radial strain. Longitudinal strain has been used for the early detection of LV impairment in a variety of cardiac diseases. Moreover, longitudinal strain changes are prognostic of cardiotoxicity in patients (41). One possibility is that longitudinal, radial, and circumferential strains change at a different rate (42). By measuring only at one-time point, we might have missed eventual changes in some parameters. Besides, endocardial fibers are mainly longitudinaly oriented, while mid-wall are more circumferential oriented (43). Thus, orientation and local geometry might respond differently to exercise-induced hemodynamic stress. Stewart and collaborators demonstrated that acute exercise transiently reduces longitudinal strain globally (44). In contrast, this intervention only causes reduction in the apical portion of the circumferential strain. ExTr induces physiological modification of cardiac geometry toward cavity expansion in response to repeated bouts of transient hemodynamic overload (45, 46). In our study, we did not find significant changes in cavity size or fibrotic depots. These findings suggest that the LV morphology is differently affected by ExTr under Doxo administration. Circumferential strain in the short axis is more sensitive in identifying LV remodeling (47), which seems to explain why circumferential strain was more sensitive than other strain parameters in our study. Finally, one should consider differences among species. Circumferential strain seems to represent more similar LV contractility patterns in mice and humans (48), which enhances the translational findings of our study. Notably, the circumferential strain is pointed out as more relevant to the cardio-oncology population, by its superior sensitivity to detect patients under higher risk of developing Doxo-induced cardiotoxicity (38). Certainly, regional changes along time and in response to different stimuli is an interesting topic for further studies.

The myocardial strain has also been demonstrated to precede recovery in other cardiovascular diseases. Investigating mice after myocardial infarction, speckle tracking analysis detected that improvements in cardiac strain occurred before regaining of LVEF functionality (38). In our model, we observed both a reduction of myocardial strain and LVEF under Doxo influence. ExTr normalized this parameter. Whether myocardial strain normalization by ExTr indicates a future full recovery of Dox-induced LVEF dysfunction remains to be determined.

Exercise prompts physiological stress to the cardiovascular system and, by doing so, provide an informative assessment of cardiovascular function (38). McKillop and coworkers, more than three decades ago, demonstrated that, by adding exercise response to a prediction model based on LVEF, the sensitivity nearly doubled, reaching 100% chance to detect Doxo-induced cardiotoxicity compared to resting LVEF alone (49). Others also suggested the inclusion of exercise tests as an important tool, especially to avoid underestimation of subclinical dysfunction (49, 50) or to distinguish from pre-existent coronary artery disease (51). Indeed, ongoing clinical trials are investigating whether exercise intolerance and cancer fatigue can predict cardiotoxicity and cardiovascular events in the next decade (NCT02791581, ClinicalTrials.gov). Here, we showed that ExTr improved exercise capacity, preventing Doxo-induced exercise intolerance and, hence, counteracting cancer-related fatigue. Interestingly, a significant correlation between myocardial contractility and exercise capacity was observed (Figure 6D). Of note, exercise capacity is a predictor of survivorship across different types of cancer, some of them traditionally treated with Doxo (52–54). Hence, our study suggests that ExTr improves cardiovascular fitness and counteract Doxo-induced cardiotoxicity.

### Study Limitations

We studied male mice as it has been reported a protective role of the female sex in Doxo-induced cardiotoxicity (53). Hence, the implications of the present study may offer limited insights for the female population. The fact that we narrowed down our molecular analysis to specific, previously selected pathways, may have limited the strength of our mechanistic conclusions. Also, we studied low-to-moderate intensity aerobic exercise. However, it is possible that higher exercise dose would elicit superior effects (15, 55).

## Conclusion

Our findings suggest that low-to-moderate intensity aerobic ExTr program performed concomitantly with Doxo treatment does not prevent Doxo-induced LVEF reduction but attenuates cardiac atrophy, preserves myocardial strain, and enhances exercise tolerance. Therefore, ExTr can be a valuable approach to alleviate Doxo-induced cardiotoxicity.

## Data Availability Statement

The raw data supporting the conclusions of this article will be made available by the authors, without undue reservation.

## Ethics Statement

The animal study was reviewed and approved by Ethics Committee on Animal Use (CEUA) of the University of São Paulo Medical School (FMUSP).

## Author Contributions

IG-S, CN, RC, and AC: designed experiments. IG-S, CJ, and CP: performed experiments. IG-S and CJ: analyzed data. PB, EO, RC, AC, and CN: provided laboratory space, reagents, and technical support. IG-S and CN: wrote the manuscript. IG-S, CJ, CP, PB, EO, RC, AC, and CN: edited the manuscript. CN: supervised the study. All authors: contributed to the article and approved the submitted version.

## Conflict of Interest

The authors declare that the research was conducted in the absence of any commercial or financial relationships that could be construed as a potential conflict of interest.
